# A scoping review of ICD-11 adjustment disorder research

**DOI:** 10.1080/20008198.2017.1421819

**Published:** 2018-01-18

**Authors:** Evaldas Kazlauskas, Paulina Zelviene, Louisa Lorenz, Soledad Quero, Andreas Maercker

**Affiliations:** ^a^ Department of Clinical and Organizational Psychology, Vilnius University, Vilnius, Lithuania; ^b^ Department of Psychology, Division of Psychopathology and Clinical Intervention, University of Zurich, Zurich, Switzerland; ^c^ Department of Basic and Clinical Psychology and Psychobiology, Universitat Jaume I, Castellon de la Plana, Spain

**Keywords:** Adjustment disorder, ICD-11, review, validation, assessment, treatment, Trastorno adaptativo, CIE 11, validación, evaluación, tratamiento, 适应障碍, ICD-11综述, 验证, 评估, 治疗, • Significant revisions of adjustment disorder definition are included in ICD-11.• Findings from the validation studies of the ICD-11 proposals are ambiguous.• A new measure of the ICD-11 adjustment disorder is available and is currently undergoing validation in various samples.• Low intensity self-help interventions for the ICD-11 adjustment disorder show promising outcomes.

## Abstract

**Background**: Adjustment disorder (AjD) is one of the most used mental disorder diagnoses among mental health professionals. Important revisions of the AjD definition in the 11th edition of the International Classification of Diseases (ICD-11) are proposed. AjD is included in a chapter of disorders specifically associated with stress in ICD-11.

**Objective**: This paper aims to review recent developments in ICD-11 AjD research, and to discuss the available ICD-11 AjD diagnosis validation studies, AjD measures, treatment studies, and outline the future perspectives for AjD research and clinical practice.

**Methods**: In total, 10 empirical studies of AjD ICD-11 were identified and included in this review. We searched for studies in Embase, PubMed, PsycINFO, Scopus, PILOTS, SocINDEX, and via additional search by contacting authors of published empirical studies and reference screening.

**Results**: Review of the studies revealed a lack of validation studies of the ICD-11 AjD symptom structure. AjD validation study findings are ambiguous, and there is still little support for the proposed two symptom structure of AjD for the ICD-11. A self-report AjD measure ‘Adjustment Disorder New Module’ (ADNM) based on the ICD-11 definition has been developed and used in all 10 reviewed studies. Two self-help interventions have been developed for the ICD-11 AjD, and findings from these studies indicate that self-help low-intensity cognitive-behavioural interventions, delivered via bibliography or internet-based, might be effective treatment of AjD.

**Conclusions**: The AjD definition in ICD-11 with a description of a new symptom profile facilitates AjD measurement and AjD-focused treatment developments. More studies and insights from clinical practice are needed to move the field of AjD research and practice forward.

## Abstract (es)

**Contexto**: El trastorno de adaptación o adaptativo (TA) se encuentra entre los diagnósticos de trastorno mental más usados entre los profesionales de la salud mental. Se proponen revisiones importantes de la definición de T. adaptativo de la 11ª edición de la Clasificación Internacional de Enfermedades (ICD 11). El T. adaptativo está incluido en la CIE 11 en un capitulo de trastornos específicamente asociados con el estrés.

**Objetivo**: Este articulo pretende revisar desarrollos recientes sobre la investigación del T. adaptativo en la CIE 11 así como discutir los estudios de validación del diagnostico del mismo para la CIE 11. También medidas del T. adaptativo, estudios sobre tratamiento y la delineación de perspectivas futuras de la investigación sobre el T. adaptativo y la práctica clínica.

**Métodos**: En total, fueron identificados e incluidos en esta revisión, 10 estudios empíricos sobre el T. adaptativo para la CIE 11. Revisamos la presencia de trabajos en las bases Embase, PubMed, PsycINFO, Scopus, PILOTS, SocINDEX y a través de una vía adicional contactando a los autores de estudios empíricos publicados. También se realizó una discriminación de referencias bibliográficas.

**Resultados**: La revisión de los estudios revelaron una falta de estudios de validación de la estructura de síntomas para el T. adaptativo en la CIE 11. Los hallazgos de los estudios de validación son ambiguos, y todavía hay un soporte escaso para la propuesta estructura en dos síntomas del T. adaptativo en la CIE 11. Ha sido desarrollado un autoreporte de la medida del T. adaptativo denominada ‘Nuevo modulo del T. adaptativo’ (ADNM) basada en la definición de la CIE 11. Ha sido usada en todos los 10 trabajos revisados. Han sido desarrolladas dos intervenciones de autoayuda para el T. adaptativo de la CIE 11 y los hallazgos obtenidos por estos estudios indican que las intervenciones de autoayuda de baja intensidad, cognitivo – conductuales, administradas vía bibliografía o por Internet, pueden ser un tratamiento efectivo para el T. adaptativo.

**Conclusiones**: La definición de T. adaptativo en la CIE 11, con la descripción de un nuevo perfil sintomatológico, facilita la medida del T. adaptativo y los desarrollos del tratamiento focalizado en el trastorno. Se necesitan más estudios y experiencias desde la práctica clínica para impulsar hacia delante el campo de la investigación y práctica del diagnóstico del T. adaptativo.

## Abstract (zh)

**背景：**适应障碍（AjD）是精神健康专家最常用的精神疾病诊断。在《第11版国际疾病分类》（ICD-11）中提出了对 AjD 定义的一些重要修订。AjD在 ICD-11中被涵括在与应激有关障碍的章节中。

**目标：**本文旨在综述最近关于ICD-11 AjD的研究进展，同时对已有的ICD-11 AjD诊断验证、测量和治疗研究进行讨论，并提出AjD研究和临床实践的未来前景。

**方法：**共有10个关于ICD-11 AjD 的实证研究被检索识别，并包括在本综述中。我们在 Embase，PubMed，PsycINFO，Scopus，PILOTS，SocINDEX中检索研究，也通过联系已发表的实证研究的作者和参考文献筛选来作为额外补充检索。

**结果：**这些研究的综述显示ICD-11 AjD 症状结构缺乏验证研究。AjD验证研究发现是模糊不清的，而且ICD-11中提议的AjD症状结构的支持较少。一个基于ICD-11定义的AjD自评测量《适应障碍新模块（ADNM）》 被开发并使用在所有10个研究中。两种自助干预方法被用于ICD-11 AjD，研究结果表明通过参考书目或者网络进行的自助式的低强度认知行为干预可能对AjD有效。

**结论：**ICD-11的AjD定义描述了一个新的症状侧写，促进了AjD的测量和治疗的发展。需要更多研究和来自临床的见解来推动AjD领域的研究和实践。

Adjustment disorder (AjD) is one of the most used mental disorder diagnoses among psychologists and psychiatrists according to worldwide surveys of mental health professionals (Evans et al., 2013; Reed, Mendonça Correia, Esparza, Saxena, & Maj, 2011). AjD is a mental disorder with serious implications; in particular, it is highly associated with suicidality (Casey, Jabbar, O’Leary, & Doherty, 2015). A recent longitudinal study revealed that AjD is a common condition after traumatic injuries (O’Donnell et al., 2016). Still, AjD remains largely neglected in research, in contrast to other stress-related disorders such as posttraumatic stress disorder (PTSD) or complex PTSD (Brewin et al., 2017).

The AjD definition has passed revisions in International Classification of Diseases (ICD). The first attempt to define AjD in ICD was the inclusion of ‘transient situational disturbances’ diagnosis in the eighth edition of ICD in 1965, and ‘adjustment reaction’ in the ninth edition of ICD in 1975. Under the name of ‘adjustment disorder’ this diagnosis was included in the 10th edition of the ICD, and was defined as a subjective distress or emotional disturbance following a significant life change or stressful life event (WHO, 1992). The ICD-10 AjD definition described potential stressors, and included symptoms of depressed mood, anxiety, or worry in addition to functional impairment following a stressor. Important revisions of the AjD definition are in the 11th edition of the ICD (ICD-11). AjD is included in the new chapter of ‘Disorders Specifically Associated with Stress’ in ICD-11, along with other stress-related disorders, such as PTSD, complex PTSD, and prolonged grief disorder (PGD) (Maercker et al., 2013).

The main changes of AjD description in ICD-11 (ICD-11 AjD) in contrast to the ICD-10 definition of AjD is the clear definition of symptoms (Maercker et al., 2013). AjD is defined in ICD-11 as a maladaptive reaction, which usually emerges within one month of a significant life-stressor, such as illness, family or partnership problems, job-related issues, or financial difficulties. Two symptoms constitute AjD: (1) preoccupation with a stressor or its consequences; (2) failure to adapt (Maercker et al., 2013). Preoccupation with a stressor is associated with recurring distressing thoughts about the stressor, constant worry about the stressor, or rumination about the stressor. Failure to adapt describes a generalized stress-response (e.g. sleep disturbances or concentration problems) that results in significant impairment in social, interpersonal, occupational, educational, or other significant areas of functioning (Maercker et al., 2013). A recent update of the AjD definition in ICD-11 included exclusion criteria (WHO, 2017). AjD can be diagnosed only if symptoms do not reach sufficient specificity or severity of other mental disorder, similar to the DSM-5 AjD definition (American Psychiatric Association, 2013). AjD usually resolves itself within six months, unless the duration of the stressor is longer (Maercker et al., 2013).

This paper aims to review recent developments in the ICD-11 AjD research, and to provide up-to-date knowledge about the ICD-11 AjD for researchers and clinicians. We discuss ICD-11 AjD validation studies, AjD measures, treatment studies of AjD, and future perspectives for AjD research and clinical practice in our paper.

## 1. Method

We followed guidelines for scoping reviews in extracting and reporting data (Levac, Colquhoun, & O’Brien, 2010). The main question for this study was to identify and review the empirical studies of the ICD-11 AjD, including ICD-11 AjD symptom structure validation studies, ICD-11 AjD measures, and ICD-11 AjD treatments to evaluate the available evidence for the ICD-11 AjD symptom structure, measures, risk factors, and treatments. Our inclusion criteria for this review were: (1) AjD symptoms measured using ICD-11 diagnostic criteria, (2) the empirical data reported, and (3) the study was peer-reviewed and published.

We searched for published ICD-11 AjD studies using the keywords ‘adjustment disorder’ and ‘ICD-11ʹ in abstracts and titles of articles indexed in Embase, PubMed, PILOTS, PsycINFO, Scopus, and SocINDEX databases in August 2017. The search in the databases revealed 25 articles. Two reviewers independently screened the abstracts of the extracted articles. After screening of titles and abstracts, we identified six published ICD-11 AjD empirical studies meeting our inclusion criteria. In total, 19 papers from the database search were excluded from further analysis because they were theoretical, study protocol papers, or other articles without reported empirical data. After the database search we screened the reference lists, and contacted authors of the extracted studies to enquire about other AjD published papers or articles in press. We identified four more articles in addition to the six studies identified through database search, which met inclusion criteria for this review.

In total, 10 empirical studies were included in the full-text analysis. Data from the 10 full-text articles were extracted by the two reviewers. Sample descriptions, methods, and main findings of the 10 published AjD ICD-11 studies included in this review are presented in Table 1. All participants in the reviewed studies were adults, and samples ranged from a single case study to national representative samples (*N* = 2512).

**Table 1.** Empirical studies of ICD-11 adjustment disorder (*N* = 10).

Authors	Study focus	Study design	Sample and stressor	Sample size	Country	Measures	Main findings
Bachem et al. (2016)	Measure validation	Secondary analysis of the previous RCT	Clinical sample diagnosed with DSM-IV AjD, various stressors	190	Republic of South Africa	ADNM-29, HAM-A, MADRS, SDS	AjD symptoms decreased significantly in treatment group; convergent and divergent validity was supported using HAM-A, SDS, MADRS
Bachem and Maercker (2016)	Treatment	RCT	Burglary victims	54	Switzerland	ADNM-20, CSQ-8, DASS-21, PTSD-ICD-11	AjD symptoms decreased significantly in treatment group; between-group effect size at three-month follow-up compared to waiting list for AjD preoccupation was *d* = 0.67, failure to adapt *d* = 0.34
Eimontas et al. (2017)	Treatment	RCT	Self-referred with high levels of AjD symptoms, various stressors	1077	Lithuania	ADNM-8, WHO-5	Symptoms of AjD decreased significantly with moderate effect sizes *d* = 0.53–0.64; additional support from therapist did not significantly improve the outcomes
Glaesmer et al. (2015)	Symptom structure	Cross-sectional	Representative national sample, various stressors	2512	Germany	ADNM-20	Six-symptom first-order correlated AjD CFA model had the best fit with two core AjD symptoms: preoccupation, failure to adapt, and four additional symptoms: Avoidance, depression, anxiety, and impulsivity
Horn and Maercker (2016)	Predictors of AjD	Dyadic	Couples exposed to various major stressors over the last 12 months	146	Switzerland	ADNM-8, CES-D, ERQ, IER, RSQ	Dyadic regression analysis revealed importance of interpersonal emotion regulation strategies on AjD
Lorenz et al. (2017)	Symptom structure	Cross-sectional	Individuals who lost their jobs during the last nine months prior to the study	333	Switzerland	ADNM-20, BSI-18, SFQ, OcSe, SOC-R	CFA showed that unrestricted bifactor model with a dominant general AjD factor consisting of preoccupation, failure to adapt, avoidance, affective reaction and impulsivity provided the best fit of data
Lorenz et al. (2016)	Measure validation	Cross-sectional	Burglary victims	80	Switzerland	ADNM-20, DASS-21, PTSD-ICD-11	Cluster analysis revealed three groups of individuals with low, moderate, and high AjD symptoms; cut-off score of the ADNM-20 was proposed
Maercker et al. (2015)	Treatment	Case study	Burglary victims	1	Switzerland	ADNM-6, DAAS-21	AjD symptoms decreased in AjD self-help intervention study
Mahat-Shamir et al. (2017)	Predictors of AjD	Cross-sectional	Individuals after the shooting attack in Tel Aviv	379	Israel	ADNM-20, PCL-C	Previous exposure to stressful or traumatic events during the past month were significantly positively associated with AjD; physical proximity to the traumatic event was a significant predictor for PTSD, but not for AjD
Zelviene et al. (2017)	Symptom structure	Cross-sectional	Representative national sample, various stressors over the last two years	831	Lithuania	ADNM-20	Two-factor AjD structure with preoccupation and failure to adapt symptoms was supported using CFA

ADNM = Adjustment Disorder New Module; BSI-18 = Brief Symptom Inventory, Short Form; CES-D = Center for Epidemiological Studies–Depression; CFA = Confirmatory Factor Analysis; CSQ-8 = Client Satisfaction Questionnaire; DASS-21 = Depression Anxiety Stress Scales; ERQ = Emotion Regulation Questionnaire; HAM-A = Hamilton Anxiety Rating Scale; IER = Interpersonal Emotion Regulation; LCA = Latent Class Analysis; MADRS = Montgomery–Asberg Depression Rating Scale; OsCe = Occupational Self-Efficacy Scale; PCL-C = Post-traumatic Stress Disorder Checklist–Civilian Version; PTSD-ICD-11 = ICD-11 Post-traumatic Stress Symptoms; RSQ = Response Style Questionnaire; SFQ = Social Functioning Questionnaire; SDS = Sheehan Disability Scale; SOC-R = Sense of Coherence Scale–Revised; WHO-5 = Well-being Index.

## 2. Results

### 2.1. ICD-11 AjD measures

The self-report scale ‘Adjustment Disorder New Module’ (ADNM) was used in all 10 reviewed studies for measuring ICD-11 AjD symptoms. This AjD measure was proposed prior to the ICD-11 proposals with an introduction of the ADNM in 2007 (Einsle, Köllner, Dannemann, & Maercker, 2010; Maercker, Einsle, & Köllner, 2007). The ADNM has two parts. The first part of ADNM includes a list of potential acute or chronic stressful life-events and the second part of the ADNM is a list of AjD symptoms. The stressor list of the ADNM was used in nine reviewed studies. However, the number of stressors in the first part of the ADNM ranged considerably across different studies: 10 stressors (Bachem & Maercker, 2016; Bachem, Perkonigg, Stein, & Maercker, 2016), 13 stressors (Horn & Maercker, 2016), 15 stressors (Zelviene, Kazlauskas, Eimontas, & Maercker, 2017), 16 stressors (Glaesmer, Romppel, Brähler, Hinz, & Maercker, 2015), 17 stressors (Eimontas et al., 2017), and 19 stressors (Lorenz, Bachem, & Maercker, 2016).

There are also variations in the number of the symptom items in the second part of the ADNM in the reviewed AjD ICD-11 studies. Four versions of ADNM were used in studies: the brief 6-item ADNM-6, 8-item ADNM-8, 20-item ADNM-20, and 29-item ADNM-29. Six of the reviewed studies used the 20-item ADNM-20 scale, which included the 20 symptom items comprising six subscales: preoccupation (four items), failure to adapt (three items), avoidance (four items), depression (three items), impulsivity (three items), anxiety (two items) (Glaesmer et al., 2015; Lorenz, Hyland, Perkonigg, & Maercker, 2017; Lorenz et al., 2016; Mahat-Shamir et al., 2017; Zelviene et al., 2017). The 29-item ADNM-29 was used in one study (Bachem et al., 2016). However, the authors of this study reduced number of items to 20 in the analysis of data. A brief 8-item ADNM-8 symptom scale with a two main AjD symptoms: failure to adapt (four items), and preoccupation (four items) was used in two studies (Eimontas et al., 2017; Horn & Maercker, 2016), and a short 6-item ADNM-6 was used in a case study by Maercker, Bachem, Lorenz, Moser, and Berger (2015). The scoring of the ADNM is the sum of items for the total scale and subscales in all versions of the ADNM, and the 4-point Likert scale with the frequency of symptoms (1 = never, 2 = rarely, 3 = sometimes, 4 = often) is used.

Two studies particularly focused on testing the validity of the ADNM-20. Lorenz et al. (2016) analysed psychometric properties of the ADNM-20 among Swiss burglary victims (*N* = 80) (Lorenz et al., 2016). Cluster analysis identified three groups of individuals with low, moderate, and high AjD symptoms in this study. However, the specificity of the ADNM-20 was rather low (74%), and positive predictive value for AjD diagnosis was poor (57%) (Lorenz et al., 2016). The ADNM-20 sensitivity to change, and convergent and discriminant validity, was tested in psychopharmacological (Etifoxine and Alprazolam) AjD treatment study in South Africa (Bachem et al., 2016). The ADNM-20 was administered to outpatients diagnosed with the DSM-IV AjD diagnosis (*N* = 190). The data of this study was collected at four time-points: baseline, seven-day, 28-day, and 38-day follow-up. At baseline, 78% of individuals diagnosed with DSM-IV AjD diagnosis were also classified as having AjD based on the ADNM scores. AjD symptoms measured with ADNM-20 significantly declined during treatment, parallel to the decline of the depression (*r* = .13–.30), anxiety symptoms (*r* = .18–.31), and functional impairment (*r* = .18–.47) measured with other measures (Bachem et al., 2016).

### 2.2. ICD-11 AjD symptom structure validation

Three studies tested the validity of the ICD-11 AjD symptom structure. Glaesmer et al. (2015) tested three AjD symptom structure models using a Confirmatory Factor Analysis (CFA) in a large German general population sample (*N* = 2512). Six symptoms of AjD: preoccupation, failure to adapt, avoidance (Av), depression (De), anxiety (Ax), and impulsivity (Im) comprising of 2–4 items each were included into the CFA analysis. CFA supported the general AjD factor, with the best fit of the first-order six-factor correlated CFA model, in contrast to a first-order single-factor CFA model, and second-order single-factor CFA model (Glaesmer et al., 2015). However, the factors in the six-factor model were highly correlated and interpretation of this model is limited. Furthermore, the same study identified three quantitatively differing latent classes using a Latent Class Analysis (LCA) approach with low symptom, mild symptom, and moderate symptom severity (Glaesmer et al., 2015).

The other AjD symptom structure validation study was conducted in the Lithuanian national representative sample (*N* = 831) by Zelviene et al. (2017). This study explored the structure of AjD symptoms and found that the two-factor AjD CFA model consisting of the two symptoms preoccupation and failure to adapt fitted the data best (Zelviene et al., 2017). However, the authors of this study also found that an alternative AjD symptom structure CFA model with additional Av, De, Ax, and Im symptoms also had a rather good model fit, and concluded that the AjD structure might need additional symptoms in the future to capture the full clinical picture of AjD (Zelviene et al., 2017).

A recent study in a Swiss sample of individuals affected by involuntary job loss (*N* = 333) provided a comprehensive assessment of the latent structure of AjD symptomatology using CFA (Lorenz et al., 2017). Seven AjD factor structure models were tested and compared: (1) five first-order AjD factor models with preoccupation, failure to adapt, affective reaction, Av, and Im factors; (2) one second-order factor with the same five factors included in one AjD second-order factor; and (3) two bifactor models with one general AjD factor in addition to the first-order factor model (Lorenz et al., 2017). An unrestricted bifactor model with a dominant general AjD factor provided the best fit. The authors suggest the plausibility of a unidimensional solution that should be subject to further studies.

### 2.3. ICD-11 AjD predictors

Two studies analysed factors associated with symptoms of ICD-11 AjD (Horn & Maercker, 2016; Mahat-Shamir et al., 2017). Seventy-three couples with exposure to a major stressor over the last 12-months participated in a study which explored the role of interpersonal emotion regulation strategies on adjustment disorder (Horn & Maercker, 2016). The study found that interpersonal co-brooding and depressive symptoms were significantly associated with symptoms of AjD, supporting the importance of interpersonal social factors in AjD (Horn & Maercker, 2016).

The second study explored AjD predictors following the terror attack in Tel Aviv (*N* = 379) (Mahat-Shamir et al., 2017). Previous traumatic exposure was a significant predictor of PTSD and AjD, while previous exposure to stressful events during the past month was significant predictor for AjD, but not PTSD. Physical proximity to the terror event was a significant risk factor for PTSD, but not for AjD (Mahat-Shamir et al., 2017). Age was a significant predictor of AjD in this study.

### 2.4. Intervention studies of ICD-11 AjD

Three studies reported outcomes of cognitive behavioural (CBT) self-help interventions for ICD-11 AjD (Bachem & Maercker, 2016; Kazlauskas, Zelviene, & Eimontas, 2017; Maercker et al., 2015). One of the reviewed studies included a sample of patients in psychopharmacological treatment (Bachem et al., 2016). However, the main aim of this study was validation of the ADNM instrument and not treatment outcomes. Furthermore, this study used DSM-IV classification for AjD diagnosis (Bachem et al., 2016).

A group at the University of Zurich developed a self-help printed manual for burglary victims which could be delivered without therapist support (Bachem & Maercker, 2016; Maercker et al., 2015). This low-intensity intervention has a specific focus on the ICD-11 AjD core symptoms: preoccupation and failure to adapt. There are four modules of exercises in this intervention: ‘Sense of self’, ‘Coping’, ‘Activation’, and ‘Relaxation’. A case study indicated feasibility of this approach with a significant decline of AjD symptoms at the three-month follow-up (Maercker et al., 2015). A waiting list RCT study with three-month follow-up (intervention group *n* = 30, waiting list *n* = 25) revealed promising findings, with between-group effect size of *d* = 0.67 for preoccupation, and *d* = 0.34 for failure to adapt symptoms (Bachem & Maercker, 2016). The authors of the intervention proposed that an e-health self-help approach might be suitable for an AjD (Maercker et al., 2015).

The second AjD intervention is the self-help unguided internet-delivered Brief Adjustment Disorder Intervention (BADI) developed by a Vilnius University group in Lithuania (Eimontas et al., 2017; Skruibis et al., 2016). The BADI intervention has four modules with three exercises in each of the modules, including relaxation, daily activity planning, interpersonal relationship conflict management, and mindfulness. Participants of this intervention have access to all the modules and exercises after registration online, and can freely choose which exercises and how often they want to use it. This low intensity ICD-11 AjD intervention can be used without therapist support. Findings of the recent study (*N* = 1077) revealed moderate effect sizes (*d* = 0.53–0.64) of the BADI intervention on AjD symptoms in this study (Eimontas et al., 2017). Furthermore, additional psychologist support was not contributing significantly to the outcomes of the intervention, indicating that internet-delivered self-help AjD interventions could be considered as a promising alternative in the treatment of AjD (Eimontas et al., 2017).

## 3. Discussion

Proposals to update the definition of AjD for ICD-11 (Maercker et al., 2013) received attention in research since the introduction of new AjD symptoms in 2013. We identified 10 empirical ICD-11 AjD studies in our review with adult samples from Germany, Lithuania, Israel, the Republic of South Africa, and Switzerland. These studies analysed the AjD factor structure, measurement validity, risk factors for AjD, and outcomes of AjD interventions.

To the best of our knowledge, the only ICD-11 based measure currently available is the Adjustment Disorder New Module (ADNM) (Einsle et al., 2010) which was used in all 10 reviewed studies. The validity of the ADNM has been tested in two studies (Bachem et al., 2016; Lorenz et al., 2016). The ADNM measure, however, also includes symptoms that are associated with a previous definition of AjD in ICD-10, such as anxiety and depression symptoms. Furthermore, there is a big variation in the number of the ADNM items across all the studies, limiting comparison of the findings. Further validation of the ADNM, with test-retest reliability analysis and cross-cultural studies, or the development of a new more appropriate measure is needed.

The findings on the AjD symptom structure as proposed for the ICD-11 are ambiguous. We found only three ICD-11 AjD symptom structure validity studies using the CFA approach. The Lithuanian study supported a two-factor AjD symptom structure (Zelviene et al., 2017), but an alternative model with more symptoms was reported as feasible. The study of a German sample found that a correlated six-factor solution was the most applicable (Glaesmer et al., 2015). A recent Swiss study supported the unidimensional structure of AjD symptoms (Lorenz et al., 2017). So far, empirical data do not provide enough support for the ICD-11 definition of AjD symptom structure. None of the analysed studies attempted to compare ICD-10 and ICD-11 AjD diagnostic criteria. Further studies of AjD symptom structure in other samples, and particularly in clinical groups, are needed.

The study of AjD risk factors reveals the importance of the nature of stressful event. In line with proposals for the ICD-11, PTSD was predicted by the previous traumatic experiences, but not by the exposure to a stressful event in the past month. AjD was predicted both by stressful experiences and previous trauma exposure in the same study (Mahat-Shamir et al., 2017). Furthermore, demographic characteristics, such as age, and interpersonal factors, such as emotional regulation, seem to be important for AjD (Horn & Maercker, 2016; Mahat-Shamir et al., 2017). Furthermore, initial findings indicate that AjD can also be associated with traumatic experiences (Mahat-Shamir et al., 2017; O’Donnell et al., 2016), and this should be explored in further studies.

Only a few AjD intervention studies were available prior to the ICD-11 proposals (Casey & Bailey, 2011). After the ICD-11 proposals (Maercker et al., 2013), there is still very limited data available on treatment of ICD-11 AjD. Two RCT studies indicated promising effects of the specialized CBT-based self-help interventions targeted towards symptoms of ICD-11 AjD (Bachem & Maercker, 2016; Eimontas et al., 2017). An internet-delivered approach might be suitable for treatment of AjD (Eimontas et al., 2017; Maercker et al., 2015).

## 4. Future perspectives

The position of AjD among other mental disorders remains largely unclear. Looking at AjD dimensionally from a general psychopathology factor perspective (Stephan et al., 2016), we could assume that AjD could be placed at the lower end of the psychopathology continuum. The most recent proposals for ICD-11 AjD definition place AjD as a clinical category ‘below’ other mental disorders, stating that AjD could be diagnosed only if another mental disorder is not diagnosed based on symptom intensity (WHO, 2017).

Even if AjD would be diagnosed based on an exclusion criterion when the symptoms of an individual do not fully meet criteria of another disorder, such as PTSD or depression, clinicians and researchers need measures to identify if AjD symptoms are clinically significant. A recent survey of mental health practitioners indicated that clinicians are having difficulties with ICD-11 AjD symptom identification (Keeley et al., 2016). The ICD-11 definition provides a new platform for the development of AjD symptom measures to help clinicians to deal with difficulties associated with diagnosing AjD. The available ADNM measure needs further validation in clinical samples and in a cross-cultural context to be truly useful for clinicians. Structured diagnostic interview of ICD-11 AjD symptoms is needed. Further development of AjD measures will be a challenging task, complicated by the diversity of life-stressors which cause AjD. It could be that different cut-off scores of measures for AjD diagnosis across various population and different stressors will be developed. Diagnostic research criteria for the ICD-11 AjD diagnosis could facilitate research in this field and should be developed in the near future.

The optimal treatment approach of AjD remains largely unclear. There are no AjD evidence-based treatment guidelines available so far. AjD symptoms can last for six months or more if the stressor persists, based on the ICD-11 definition. We could expect diverse symptom trajectories in association with stressful situational developments. AjD symptoms might become more severe if a stressor becomes more severe, and AjD symptoms could decrease if the stressful situation is resolved, e.g. an individual finds a job after a prolonged period of unemployment. If AjD symptoms persist, health care providers need to take the responsibly of providing the best treatment available. We expect that the ICD-11 AjD definition with a proposed new symptom profile can facilitate AjD-focused specialized treatment developments. The first developments of ICD-11 AjD-focused interventions from Switzerland and Lithuania show encouraging outcomes. CBT appears to be promising for treatment of AjD based on the available evidence, however, CBT is the only approach tested so far in the ICD-11 AjD treatment. Low intensity self-help AjD interventions, and internet-delivered AjD interventions could be the most cost-effective solutions for health care and useful for clinicians.

The two mental disorders diagnostic classifications are used worldwide in healthcare: DSM and ICD. We focused on the ICD-11 AjD studies in our review. However, the DSM-5 definition of AjD (American Psychiatric Association, 2013) differs significantly from the AjD description in the ICD-11 (WHO, 2017). The major difference of the AjD definition in DSM-5 is a broader definition of AjD without description of clearly defined symptoms and inclusion of AjD subtypes in contrast to the ICD-11. A study by Bachem et al. (2016) found 78% concordance in the DSM-IV and the ICD-11 AjD diagnosis, indicating that the DSM and the ICD have similarities in AjD criteria. However, incongruences in major diagnostic classifications in the future could result in a diverse understanding of AjD across different countries and professionals, depending on their use of the DSM versus the ICD in clinical practice and research.

We conclude that updates of AjD definition in ICD-11 could significantly contribute to the advancement of AjD understanding. Still, there is a need for more studies and insights from clinical practice to move the field of AjD research and practice forward.
